# Cooperative Distributed Uplink Cache over B5G small cell networks

**DOI:** 10.1371/journal.pone.0299690

**Published:** 2024-04-04

**Authors:** Waheed Ur Rehman, Mubarak Mohammed Al-Ezzi Sufyan, Tabinda Salam, AbdulRahman Al-Salehi, Qazi Ejaz Ali, Abdul Haseeb Malik

**Affiliations:** 1 Department of Computer Science, University of Peshawar, Peshawar, Pakistan; 2 Department of Networks and cyber security, AlJanad University of Science and Technology, Taiz, Yemen; 3 Department of Computer Science, Shaheed Benazir Bhutto Women University Peshawar, Peshawar, Pakistan; 4 Department of Computer Science, COMSATS University Islamabad (CUI), Islamabad Campus, Islamabad, Pakistan; RMIT University, AUSTRALIA

## Abstract

The emergence of content-centric network has resulted in a substantial increase in data transmission in both uplink and downlink directions. To tackle the ensuing challenges of network congestion and bottlenecks in backhaul links within Beyond Fifth Generation (B5G) networks, data caching has emerged as a popular solution. However, caching for uplink transmission in a distributed B5G scenario poses several challenges, including duplicate content matching and users’ obliviousness about cached contents. Furthermore, it is important to maximize available space by caching the most popular contents in a distributed manner. In this paper, we propose two schemes for uplink transmission in distributed B5G SCNs. The first scheme focuses on content matching to eliminate duplicate contents among distributed caches, while the second scheme redistributes un-duplicated cached contents among distributed caches based on their available space and content’s size. These approaches aim to enhance energy and spectral efficiency by reducing unnecessary uploads and optimizing distributed content caching, in addition to improve the content delivery. The analysis shows that the proposed schemes outperform the existing schemes by improving the cache hit ratio, cache hit probability, overall distributed cache efficiency, and diversity by 29.17%, 74.89%, 24.17%, and, 80%, respectively. Furthermore, the average throughput, Spectrum Efficiency (SE), and Energy Efficiency (EE) of the access network is improved by 17.78%, 18%, and 78%, respectively. Besides that, the EE and SE of both the sidehaul and backhaul links of the SBSs are also improved.

## 1 Introduction

In recent years, there has been a significant increase in data production and demand from end-users, leading to exponential growth in data streaming. It is expected that the number of internet users will reach 5.3 billion by 2023 [1]. This growth has profound implications for the network capacity of Beyond 5th Generation (B5G) networks, including challenges such as traffic load, congestion, latency, content popularity, energy consumption, and spectrum usage, as well as storage and content delivery [2–6].

To address these challenges, small cell technology is being widely advocated in 5G and B5G networks. However, implementing small cells introduces additional complexities, such as varying data requirements of users based on time and location, and potential bottlenecks in the uplink direction due to the improved data transmission rate in small cell networks (SCNs) [7–9].

Another approach to tackle the data explosion is by implementing caching in the network (caching in the air). Some significant researches have been focused on distributed caching in the cellular networks such as SCNs, particularly in the context of downlink caching [10, 11], while in the context of the uplink caching [12–16], which is the primary focus of this paper.

For downlink transmission, the authors in [10], presented “eNCache” as a cooperative caching method to perform the cooperation among the routers in the neighborhood to the content delivery via on-path caching concept. When, the eNCache handled and decided at every router to decide the interaction between routers and neighbors by providing an extension to the Named Data Networking Interest packet structure, the content delivery improved, in addition to enhance the cache hit ratio and diversity. While the authors in [11], proposed Dijkstra algorithm based cooperative caching strategy for UAV-assisted edge computing system to improve the quality of service (QoS) by reducing the content delivery delay by computing the delay of the content transmission among neighboring nodes, in addition the popular contents stored in the cache of the small-BSs to provide wireless data transmission services, that reduced the traffic load of the network and content transmission delay.

However, in the uplink direction, some researches have been presented such as, the authors in [12], proposed a novel upload cache architecture that supports parallel uploading of segmented files. Meanwhile in [13] the researchers, introduced an uplink cache system for delay-tolerant SCNs and analyzed the effectiveness of cache size for uplink caching. The authors suggested eliminating duplicated contents at the Small Base Station (SBS) by matching the hash key of file chunks after uploading the actual content. However, this approach was deemed impractical for the uplink scenario.

In the work presented by the authors in [14], they proposed a novel multiple-input multiple-output (MIMO) network architecture that incorporated a large number of Base Stations (BSs) to facilitate cache-enabled uplink transmission. One of the key contributions of that study was the introduction of the modified von Mises distribution as a popularity distribution function. By utilizing this distribution, they were able to derive the outage probability and establish a direct correlation between cache storage and outage probability. Furthermore, their observations revealed that increasing the cache storage space and network density resulted in an enhanced delivery rate.

While the papers [12–14] proposed innovative uplink cache architectures and schemes to enhance network performance, none of them considered the impact of caching on both energy efficiency (EE) and spectrum efficiency (SE), in addition to the distributed and cooperative caching. Moreover, the existing literature used SBSs [13] and BSs [14] for content matching and elimination of duplicated contents, assuming that the Mobile Stations (MSs) would be unaware of cached contents. This could result in unnecessary content uploads, which is undesirable. In contrast, in [15] introduced the Broadcast cache assist uplink (BCAU) scheme, which performs matching between attributes of cached contents and incoming content at an MS level. As a result, redundant uploading of available content(s) in the SBS cache before actual content transmission is avoided. This approach significantly improved the EE and throughput of uplink transmission over B5G-SCN.

In [16], the authors presented the cooperative distribution of the SBS cache through a framework. This framework generated thorough to create the contents list from all the SBSs and Macro Base Station (MBS) in the coverage area to assist the uplink transmission by eliminating the duplicated contents among them. Additionally, content matching at an MS was emphasized, which effectively improved energy and spectrum efficiency. Furthermore, incoming contents with large sizes were splitted, and their fractions were cached in the distributed cache that improved the cache hit ratio. However, the size of existing contents placed in the distributed cache and their effects on the distributed cache’s efficiency were not discussed.

Based on the previous discussions, the existing literature pointed out that the size of cached contents significantly demeans the acting of the distributed cache and the cooperation among the distributed cache. Contents with large/medium sizes waste more space and decrease the effectiveness of the cache until senility, which is not desirable. On the other hand, each cache also requires enough free space for new caching or their segments, which can be achieved by performing the Cache Replacement Policy (CRP) to evict some of the cached contents [17]. However, this results in more energy consumption and delays because the MS’s waiting time and connection time increase. In addition to wasting more storage and delay of content delivery.

The motivation by these challenges to put forward the novel schemes for distributed cache-enabled uplink transmission. The main aim of this paper is to enhance the efficiency of the overall distributed cache by increasing the cache hit ratio and cache hit probability, while simultaneously reducing energy consumption and delays caused by cache replacement policies. Additionally, aim to minimize wasted cache storage space and allocate suitable free space in each cache for caching new contents, ultimately leading to improve the distributed cache’s performance. That is performed by proposed two schemes, namely, Proposed Distributed Uplink Cache Scheme, and Re-Distribute Un-Duplicated Cached Content (RUCC). The first proposed scheme aims to address the challenge of elimination of the duplicated distributed cached contents and then generated the list of un-duplicated distributed cached contents [16]. While the second scheme aims to address the challenge of content segmentation with large/medium sizes into smaller sizes and redistributed the un-duplicated distributed cached contents among the distributed cache. That improved the performance and cache hit ratio, in addition to maximize available space by caching the most popular contents in a distributed manner. The main contributions of this paper are based on [16] and, summarized in the following:

- Based on the matching and finding the similarity among distributed cached contents [16]. The un-duplicated cached contents of the distributed cache are generated in the list and broadcast that list to all MSs to be used as a map for the mobiles to decide whether to upload the content(s) or not.
- Segmenting the un-duplicated distributed cached contents with a large/medium sizes into many smaller segments.
- Redistributed the smaller segments of the un-duplicated cached contents among distributed cache based on the cache free space and the size of un-duplicated cached contents.
- The performance of the proposed schemes will be evaluated the efficiency of the distributed cache in the terms of the uplink of the ratio and probability of the cache hit, overall cache efficiency, and diversity method. In addition, the throughput, SE, and EE of the access link will be evaluated too. Besides that, the merits of the proposed design will be analyzed mathematically.

The organization of this paper as follows: the system model is discussed in Section II. Section III presented the proposed scheme for the distributed uplink cache. Experimental design and evaluation are discussed in Section IV, numerical results in Section V follows conclusion in sections VI.

## 2 System model

**Fig 1:**
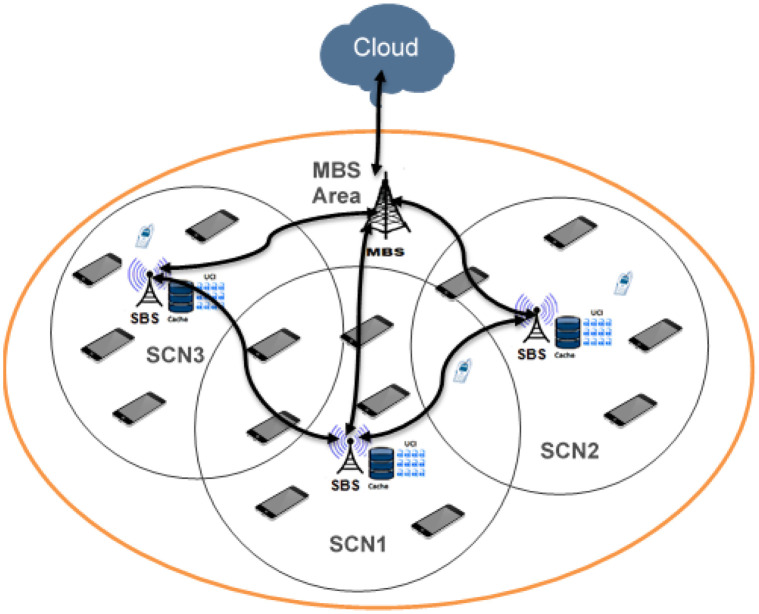
Typical scenario of distributed caching in B5G network.

In this section, the description of the system model of the distributed cache presented in details, which is consisting of Small Base Stations (SBSs),and Macro Base Station (MBS) along with the Mobile Stations (MSs). Fig 1 illustrates the overall system architecture.

The notations of this paper shown in the Table 1.

**Table 1:** Symbol notation.

Symbol	Meaning
*M*	Total small base stations
*N*	Total mobile stations
*n*	The number of MSs, which served by its respective SBS
*W*	Distributed cache capacity
*L*	Distributed cached contents-total number
*ω*	Contents of each cache-total number
*l*	Sequence number of cached content
*κ*	Attributes of content-max
*Q*	Segments of each content-total number
*m*	Total number of the SBSs, which cached duplicate content.
*P*	The attribute’s value of the cached content such as hash key.
*i*, *j*	Counters.

### 2.1 Network model

The cellular network is contained as a cloud, an MBS G, and total *M* SBSs such that $B=\{B_{j},j=1,2,...,M\}$, and *U*_*i*_ MSs such that $U=\{U_{i},i=1,2,...,N\}$ as shown in Fig 1 Time Division Duplex (TDD) is considered to be scheduled the resources of the MBS, SBSs, and MSs to provide the capability of transmitting and receiving at the same time. The system utilizes an MBS to gather information from all SBSs. The spatial distribution of the SBSs and MSs follows two separate homogeneous Poisson Point Processes (hPPP), denoted as *Φ*_*B*_ and *Φ*_*U*_, respectively. The SBSs are distributed with a density of λ_*B*_, while the MSs are distributed with a density of λ_*U*_. This independent and homogeneous distribution allows for a flexible and scalable deployment of the SBSs and MSs throughout the network. The set of MSs, which served by an SBS (*B*_*j*_) is denoted by *U*_*j*,*i*_ = {*U*_*j*,*i*_: 1 ≤ *i* < *n*, *n* < *N*}.

### 2.2 Cache model

The system incorporates a cooperative distributed cache *M* + 1 consisting of caches located at the SBSs and an MBS. This distributed cache operates as a unified entity with a combined capacity of *ω* to store a total of *L* contents. The capacity allocation for the distributed cache can be expressed as follows:

$$\begin{array}{c} W=S\left(G_{∁}\right)+\sum_{j=1}^{M}S\left(C_{B_{j}}\right),1\leq j\leq M, \end{array}$$ (1)

where $S(G_{∁})$ and $S(C_{B_{j}})$ represents the storage capacity of the cache of the MBS, and SBS, respectively.

Let T={1,2,…,T} be the slots of the time set, and t∈T is denoted to the time interval when a set of MSs uploading contents to the target SBSs. Each time slot consists of many event points and $U_{j,i}^{t}$ is the time of the connecting of an MS *U*_*j*,*i*_.

#### 2.2.1 Cache storage

Let $∁=\{C_{B_{j}}:j=1,2,\ldots ,M\}$ the list of the distributed cache of the SBSs.

The set D represents the popular contents such that $D=\{D^{l}:l=1,2,\ldots ,\omega :1\leq \omega <L\}$ are stored in the $C_{B_{j}}$. The value of *ω* represents the total count of contents cached in $C_{B_{j}}$.

The set of the cached contents of $C_{B_{j}}$ denoted by $C_{B_{j},d^{l}}=\{C_{B_{j},d^{1}},C_{B_{j},d^{2}},\ldots ,C_{B_{j},d^{\omega }}:1\leq j\leq M,1\leq l\leq \omega \}$, where the variable *l* serves as the sequential identifier for the cached content within $C_{B_{j}}$.

Referring to [18] and utilizing the Zipf distribution, the relative popularity or frequency of occurrence for each cached content item $C_{B_{j},d^{l}}$ is denoted by $Popu^{C_{B_{j},d^{l}}}$, and can be represented as;

$$\begin{array}{c} Popu^{C_{B_{j},d^{l}}}=\frac{{r^{C_{B_{j},d^{l}}}}^{-\delta }}{\sum_{l=1}^{\omega }{C_{B_{j},d^{l}}}^{-\delta }}, \end{array}$$ (2)

where the popularity rank of the $C_{B_{j},d^{l}}$ is indicated by $r^{C_{B_{j},d^{l}}}$. The value of the exponent character is denoted by *δ*.

Moreover, each $C_{B_{j},d^{l}}$ is associated with a set of attributes of each content such as hash key, name, length, size, and other relevant properties. These attributes are collectively represented by

$$\begin{array}{c} P\left(C_{B_{j},d^{l},\kappa }\right)=\{P\left(C_{B_{j},d^{l},1}\right),P\left(C_{B_{j},d^{l},2}\right),\ldots ,P\left(C_{B_{j},d^{l},\kappa }\right)\}. \end{array}$$

The $P\left(C_{B_{j},d^{l},\kappa }\right)$ will be used for performing the matching among distributed cached contents to determine and eliminate the duplicate content. While the size of content is used for segmentation each content into smaller sizes for redistributed among distributed cache. The collection of sizes of cached contents within *C*_*Bj*_ is represented by *S*(*D*) and can be defined as $S(D)=\{S(C_{Bj,d^{1}}),S(C_{Bj,d^{2}}),\ldots ,S(C_{B_{j},d^{\omega }}):1\leq j\leq M,1\leq l\leq \omega \}$.

Using [18–21], the segmented cached contents approached is considered such that the segmented is divided into *Q* segments and represented as $C_{B_{j},d^{l}}=\{{\left(C_{B_{j},d^{l}}\right)}_{1},{\left(C_{B_{j},d^{l}}\right)}_{2},\ldots ,{\left(C_{B_{j},d^{l}}\right)}_{Q}\}\forall 1\leq l\leq \omega$.

The size of each segment is determined based on two factors:

- The size of the cached content, which is denoted as $S(C_{B_{j},d^{l}})$.
- The available free space in the cache of the local Small Base Station (SBS) as well as its neighboring SBSs.

The cache’s free space of an SBS *B*_*j*_ is indicated by $C_{B_{j}}^{f_{S}}$, and it can be calculated as follows:

$$\begin{array}{c} C_{B_{j}}^{f_{S}}=S\left(C_{B_{j}}\right)-\sum_{l=1}^{\omega }S\left(C_{B_{j},d^{l}}\right), \end{array}$$ (3)

#### 2.2.2 Efficiency of a cache

The efficiency of a cache can be assessed by analyzing several key metrics. These include the cache hit ratio, which measures the proportion of requested content that is successfully retrieved from the cache. The cache miss ratio, represented, which indicates the fraction of requested content that is not found in the cache and the retrievation from the original source is needed.

According to [22, 23], the ratio of the cache hitting, and missing of each SBS $C_{B_{j}}$ are presented by $Hit_{r}^{C_{B_{j}}}$ and $Miss_{r}^{C_{B_{j}}}$, respectively and are given as:

$$\begin{array}{c} Hit_{r}^{C_{B_{j}}}=\left(\frac{Hit_{n}^{C_{B_{j}}}}{Hit_{n}^{C_{B_{j}}}+Miss_{n}^{C_{B_{j}}}}\right), \end{array}$$ (4)

$$\begin{array}{c} Miss_{r}^{C_{B_{j}}}=\left(1-Hit_{r}^{C_{B_{j}}}\right). \end{array}$$ (5)

Referring to the work in [24], the cache hit probability is referred to the likelihood of a randomly selected active MS finding its uploading content in the distributed cache. It is denoted by *hit*_*prob*_, and is given as:

$$\begin{array}{c} hit_{prob}=prob_{r}^{d^{l}}C_{prob}^{d^{l}}, \end{array}$$ (6)

where, $C_{prob}^{d^{l}}$ is the caching probability of content $C_{B_{j},d^{l}}$. $prob_{r}^{d^{l}}$ is the probability of content $C_{B_{j},d^{l}}$ according to [15].

### 2.3 Communication model

In this subsection, the uplink transmission of MSs presented in details. Each MS is served by its associated SBS based on their respective locations. The MS establishes a connection with its local SBS to initiate the upload of its content. The selection of the local SBS is determined by evaluating the Maximum Averaged Receive-Signal-Strength (Max-RSS). The Max-RSS is calculated considering factors such as association probability, handoff probability, and coverage probability, which are determined using a multi-directional path loss model and the K-means algorithm. While the coverage probability is evaluated using schemes like Maximum Received Power Association (MRPA) and Nearest BS Association (NBA) [25–27].

Referring to the works of [16, 28, 29], the capacity of uplink transmission of an MS denoted by ${ℜ}_{U_{j,i}}^{ul}$, and can be described as follows:

$$\begin{array}{c} {ℜ}_{U_{j,i}}^{ul}=B\;{\text{log}}_{2}\left(1+SINR_{U_{j,i}}\right), \end{array}$$ (7)

Here, the channel bandwidth is indicated by *B*. The Signal-to-Interference-and-Noise Ratio (SINR) of the received signal from Mobile Station (*U*_*j*,*i*_) at its serving SBS *B*_*j*_ is denoted as $SINR_{U_{j,i}}$. It can be mathematically represented as follows:

$$\begin{array}{c} SINR_{U_{j,i}}=\frac{TP_{U_{j,i}}^{ul}H_{i,j}^{ul}{∥d\left(U_{j,i},B_{j}\right)∥}^{-\alpha }}{\sum_{i\in I}I_{i}+\sigma^{2}}, \end{array}$$ (8)

Here, the transmit power of an uplink direction of MS *U*_*j*,*i*_ indicated by $TP_{U_{j,i}}^{ul}$. The term $H_{i,j}^{ul}$ corresponds to the uplink channel gain. The Euclidean norm is represented by ‖.‖. The variable *d*(*U*_*j*,*i*_, *B*_*j*_) signifies the distance between mobile station *U*_*j*,*i*_ and small base station *B*_*j*_. The path loss exponent is denoted by *α*. The noise power spectral density at the MS is represented by *σ*^2^. Lastly, *I*_*i*_ denotes the set of interfering MSs that are served by the same SBS *B*_*j*_, and *i*, *j* are denoted to the mobile station and its serving SBS, respectively.

### 2.4 Energy consumption (EC) model

Based on [30] and referring to the analysis of the previous work in [15], the energy consumption of the MS *U*_*i*,*j*_ is presented by $EC_{U_{i,j}}$, and can be expressed as,

$$\begin{array}{c} EC_{U_{i,j}}=E_{m}+E_{op}+TE_{U_{j,i}}^{ul}+E_{r}, \end{array}$$ (9)

where *E*_*m*_, *E*_*op*_, $TE_{U_{j,i}}^{ul}$, and *E*_*r*_ are energy consumed for execution matching, other operations, transmitting and receiving, respectively.

*E*_*r*_ takes a value of the energy cost of the receiving packet(s) from the serving SBS *B*_*j*_.

Whilst, the $TE_{U_{j,i}}^{ul}$ is calculated as follows: If the cache is hitting, then the $TE_{U_{j,i}}^{ul}$ is only calculated for the transmitting the Message of Target Destination (MoTD) where the MoTD contains a set of the attributes of content such as hash key and the target destination of uploading to the target SBS (*B*_*j*_), else the $TE_{U_{j,i}}^{ul}$ is calculated for transmitting the whole content.

The average energy consumption of the all mobiles is denoted by $Avg_{EC}^{U}$, and is calculated by dividing the summation energy consumption of mobiles according to (9) and the total number of mobiles *N*, and is given as,

$$\begin{array}{c} Avg_{EC}^{U}=\frac{\sum_{j=1}^{M}\sum_{i=1}^{n}\left(E_{m}+E_{op}+TE_{U_{j,i}}^{ul}+E_{r}\right)}{N} \end{array}$$ (10)

## 3 Proposed scheme for Distributed Uplink Cache

The distributed cache faces three key challenges: content duplication among caches, lack of knowledge about cached contents at an MSs, and the need to segment large or medium-sized cached contents for distributed storage. To overcome these challenges and enhance the MS’s quality of experience, an algorithm for distributed uplink caching is two schemes, namely, Distributed Uplink Cache Scheme (DulC), and Re-Distribute Un-Duplicated Cached Content (RUCC) Scheme proposed. The DulC is used to perform the matching among the distributed cached contents and eliminated the duplicated among them, and then generated the list of Un-Duplicated Content List (UDCL)to be used by second scheme. This scheme presented in Algorithm 1, utilizes the Adaptive Content Validity Period (AcVP) to validate duplicate cached contents with the assisted the Broadcast Cache Assist Uplink (BCAU) algorithm in [15] (*previous work*) to perform the matching an incoming content with the list of the distributed cached contents at the MSs level to upload only dissimilar content. Furthermore, to address the challenge of content segmentation and improve the performance and cache hit ratio, the *Re-Distribute Un-Duplicated Cached Content (RUCC) Scheme* is proposed. This scheme presented in Algorithm 2, focuses on segmenting and redistributing the cached contents to smaller sizes for efficient distributed storage.

### 3.1 Proposed Distributed Uplink Cache Scheme (DulC)

The proposed scheme consists of the following steps:

#### 3.1.1 Generating a list of distributed cached contents

Ensuring that an MS possesses a comprehensive list of cached contents is crucial to avoid nonessential uploads. This subsection provides a detailed explanation of the approach used to generate the contents list. The MBS plays a significant role in this process by creating two distinct lists: the Un-Duplicated Content List (UDCL) and the Duplicated Content List (DCL). The UDCL is primarily designed for content matching purposes, while the DCL serves the purpose of identifying and eliminating identical contents.

*3.1.1.1 Processing of Contents at MBS and SBSs.* During the preprocessing stage, a comprehensive list of the contents present in the distributed cache is generated at both the MBS and SBSs within the network. This step involves compiling all the cached contents from the various cache locations.

The list of contents of each distributed cache at the MBS/SBSs is denoted by $C_{B_{j}/G_{∁}}^{\omega \times \kappa }$. It can be mathematically represented as follows:

$$\begin{array}{c} C_{B_{j}/G_{∁}}^{\omega \times \kappa }=|\begin{array}{cccc} P_{j}^{d_{1},1} & P_{j}^{d_{1},2} & \ldots & P_{j}^{d_{1},\kappa }\\ P_{j}^{d_{2},1} & P_{j}^{d_{2},2} & \ldots & P_{j}^{d_{2},\kappa }\\ \vdots & \vdots & \vdots & \vdots \\ P_{j}^{d_{\omega },1} & P_{j}^{d_{\omega },2} & \ldots & P_{j}^{d_{\omega },\kappa } \end{array}|, \end{array}$$ (11)

where the total number of cached contents of each cache is presented by *ω* rows. The *j* is the counter of both the MBS and SBSs. The maximum number of attributes of each content is denoted by *κ*. The cached content is denoted by *d*_?_ and its properties are indicated by $P_{j}^{d_{?},\kappa }$, where *j*, *i*, *κ* are represented the serial number of MBS/SBS, cached content, and attributed of each cached content, respectively.

Then,

$$\begin{array}{c} C_{B}=\left\{C_{B_{1}}^{\omega \times \kappa },C_{B_{2}}^{\omega \times \kappa },\ldots ,C_{B_{M}}^{\omega \times \kappa }\right\}, \end{array}$$ (12)

where $|C_{B_{j}}|=M$ i.e. Eq (12) is applicable on all the SBSs, *B*_*j*_, *j* = 1, 2, …, *M*.

*3.1.2.1 Consolidated list of distributed cached contents.* An MBS will collect the lists of distributed cached contents as shown in (11). Then, an MBS applies row-wise combination function to generate a consolidated list $C_{CoD_{d}}^{L_{d}\times \kappa }$ as following:

$$\begin{array}{c} C_{CoD_{d}}^{L_{d}\times \kappa }=|\begin{array}{c} G_{∁}^{\omega \times \kappa }\\ C_{B_{1}}^{\omega \times \kappa }\\ C_{B_{2}}^{\omega \times \kappa }\\ \vdots \\ C_{B_{j}}^{\omega \times \kappa }, \end{array}|, \end{array}$$ (13)

where the total number of content of distributed cache is denoted by *L*_*d*_.

#### 3.1.2 Filtering similar contents

To eliminate duplication, the MBS undertakes a process of matching the attributes of contents from the consolidated list generated (13), utilizing the dissimilarity function (14). This function is employed to compute dissimilarity, distinguishing typical contents (*y*) from target contents (*y*^•^) [15, 31]. The dissimilarity calculation is as follows:

$$\begin{array}{c} dissim\left(y,y^{•}\right)=\frac{\sum_{P_{d}=1}^{\kappa }\partial_{y,y^{•}}^{\left(P_{d}\right)}Dis_{y,y^{•}}^{\left(P_{d}\right)}}{\sum_{P_{d}=1}^{\kappa }\partial_{y,y^{•}}^{\left(P_{d}\right)}}, \end{array}$$ (14)

The dissimilarity function, denoted as *dissim*(*y*, *y*^•^), measures the difference between the run-of-the-mill content *y* and the target content *y*^•^. The attributes of the content are represented by $\left(P_{d}\right)$, with *κ* being the maximum number of attributes. The contribution of feature $\left(P_{d}\right)$ to the dissimilarity between *y* and *y*^•^ is denoted as $Dis_{y,y^{•}}^{\left(P_{d}\right)}$, while $\partial_{y,y^{•}}^{\left(P_{d}\right)}$ is the indicator.

The similarity between *y* and *y*^•^ is calculate as follows:

$$\begin{array}{c} sim\left(y,y^{•}\right)=1-dissim\left(y,y^{•}\right), \end{array}$$ (15)

where, the similarity of contents (*y*, *y*^•^) is shown by *sim*(*y*, *y*^•^).

The list of the values of similarity is denoted by $SIM^{L_{d}\times \kappa }$. It can be mathematically represented as follows:

$$\begin{array}{c} SIM^{L_{d}\times \kappa }=|\begin{array}{ccccc} 1 & s_{1,2} & s_{1,3} & \cdots & s_{1,\kappa }\\ s_{2,1} & 1 & s_{2,3} & \cdots & s_{2,\kappa }\\ s_{3,1} & s_{3,2} & 1 & \cdots & s_{3,\kappa }\\ \vdots & \vdots & \vdots & \vdots & \vdots \\ s_{L_{d},1} & s_{L_{d},2} & s_{L_{d},2} & \cdots & 1 \end{array}|. \end{array}$$ (16)

Determining the value of the threshold of the matching $sim_{th}^{sh}$ to select the similar contents as

$$\begin{array}{c} sim=\{\begin{array}{cc} Similar, & sim\left(y,y^{•}\right)>=sim_{th}^{sh}\\ Dissimilar, & sim\left(y,y^{•}\right)<sim_{th}^{sh} \end{array}. \end{array}$$ (17)

By employing this approach, it becomes possible to identify duplicate contents present in various caches, which can then be promptly removed or evicted only from the caches of the SBSs. This ensures that there is sufficient space available to accommodate new and unique contents, optimizing the overall efficiency and performance of the distributed caching system.

#### 3.1.3 Validity of cached contents at MBS and SBSs

Once similar contents have been identified, it is crucial to assess the validity of the existing cached contents stored in the distributed cache. It is necessary to ensure that duplicate contents are retained in only one cache, while the rest are evicted.

Among the distributed cache placed at the MBS and SBSs, all duplicate contents can be stored at the MBS since it has a larger cache size. However, when dealing with duplicate contents among the SBS caches, it becomes important to evaluate their validity and determine which cache should retain the content, while others evict it.

To address this, an Adaptive Content Validity Period (AcVP) function is proposed. The AcVP function aims to calculate the probability of whether to keep or evict a content from any of the SBS caches. This function assists in making informed decisions about content placement within the distributed cache system.

The value of the (AcVP) of the cached content is denoted by $\vee_{B_{j},d^{l}}^{C}$ and can be calculated by;

$$\begin{array}{c} \vee^{C_{B_{j},d^{l}}}=RE_{B_{j}}+C_{B_{j}}^{f_{S}}+U_{j,i}+\varrho^{C_{B_{j},d^{l}}}+T_{R}^{C_{B_{j},d^{l}}}, \end{array}$$ (18)

Here, $RE_{B_{j}}$ represents the remaining energy of an SBS *B*_*j*_. $C_{B_{j}}^{f_{S}}$ denotes the available cache space of an SBS, which is determined using Eq (3). *U*_*j*,*i*_ corresponds to the total number of MSs served by an SBS *B*_*j*_. $\varrho^{C_{B_{j},d^{l}}}$ represents the popularity of the cached content, as determined by Eq (2). $T_{R}^{C_{B_{j},d^{l}}}$ signifies the remaining time for a content to remain in the cache, and it is calculated as follows:

$$\begin{array}{c} T_{R}^{C_{B_{j},d^{l}}}=E_{t}^{C_{B_{j},d^{l}}}+\tau^{C_{B_{j},d^{l}}}, \end{array}$$ (19)

where $E_{t}^{C_{B_{j},d^{l}}}$ is the content’s expire time. $\tau^{C_{B_{j},d^{l}}}$ is the time of the last hit of that content.

After performing the content similarity, the AcVP is executed among the duplicate contents. The duplicate content with a maximum value of $\vee^{C_{B_{1},d^{l}}}$ is kept in its cache, in addition, it will be added to the *Un-Duplicated Content List (UDCL)* to be un-duplicated content. The UDCL can formally be seen in (20) as,

$$UDCL^{L^{•}\times \kappa }=|\begin{array}{cccc} P_{d_{1,1}} & P_{d_{1,2}} & \ldots & PC_{d_{1,\kappa }}\\ P_{d_{2,1}} & P_{d_{2,2}} & \ldots & P_{d_{2,\kappa }}\\ \vdots & \vdots & \vdots & \vdots \\ P_{d_{L^{•},1}} & P_{d_{L^{•},2}} & \ldots & P_{d_{L^{•},\kappa }} \end{array}|,$$ (20)

where the *L*^•^ rows present the total number of un-duplicated contents of all distributed cache, which are computed as a counter during creating the list when algorithm 1 is executed.

While the remaining can be added to the *Duplicate Content List (DCL)*, and then removed/evicted for their SBSs caches. The DCL can be mathematically represented as follows:

$$DCL^{\bar{L}\times \kappa }=|\begin{array}{cc} \vee^{C_{B_{j},d^{1}}} & H(C_{(B_{j}/G),d^{l}})\\ \vee^{C_{B_{j},d^{2}}} & H(C_{(B_{j}/G),d^{2}})\\ \vdots & \vdots \\ \vee^{C_{B_{j},d^{\bar{L}}}} & H(C_{(B_{j}/G),d^{\bar{L}}}) \end{array}|,$$ (21)

where $\bar{L}$ is the total number of the duplicate contents across SBSs caches to be eliminated from their caches.

#### 3.1.4 The final list generation

Upon determining the validity of duplicate contents, the process continues to generate the final lists of duplicated and un-duplicated contents. These lists are represented by Eqs (21) and (20), respectively. The Un-Duplicated Content List (*UDCL*) serves as a useful reference for an MS, enabling them to make informed decisions regarding whether to upload content or send an MoTD instead of uploading the real content.

#### 3.1.5 Duplication elimination in the cache

The UDCL and DCL, using AcVP as depicted in (21), are shared with all the SBSs. The contents identified with low AcVP in the DCL are considered for eviction from their respective caches, denoted by $victim(\vee^{C_{B_{j},d^{l}}},C_{B_{j}},\bar{T})$, where $\bar{T}$ denotes the time of evicted content.

Moreover, each SBS *B*_*j*_ broadcasts the UDCL to all the MSs it serves, enabling content matching accordingly. Due to continuous updates in the distributed cached contents and their varying popularity caused by actions like views, uploads, shares, downloads, etc., the UDCL undergoes periodic updates and is rebroadcasted to all the MSs to maintain content consistency across the distributed cache and the MSs.

#### 3.1.6 Proposed Distributed Uplink Cache Scheme (DulC)

Based on the discussion, the DulC is proposed as shown in Algorithm 1. Each previous sections from (3.1.1) to (3.1.5) corresponds to a step of the proposed algorithm. The steps of the proposed algorithm can be summarized as follows: The proposed DulC Algorithm consists of 5 major steps. In step 1, involves the processing of distributed cached contents at an MBS. In Step 2, similarity checks are performed to identify duplicate contents. Step 3, focuses on checking the validity of the content, resulting in the generation of two lists: UDCL and DCL. Step 4, encompasses the removal of duplicate contents from the target SBSs and the dissemination of the UDCL to all the MSs. Finally, in Step 5, the MSs utilize the un-duplicated list for content matching.

**Algorithm 1** Proposed Distributed Uplink Cache Scheme (DulC)

**Input**: $G_{∁}^{\omega \times \kappa }$, B, $C_{B}=\left\{C_{B_{1}}^{\omega \times \kappa },C_{B_{2}}^{\omega \times \kappa },\ldots ,C_{B_{M}}^{\omega \times \kappa }\right\}$, U, *U*_*j*,*i*_.

**Output**: UDCL, DCL.

**Step 1**: **Initialization of cached contents at SBSs and MBS-Processing**

*L*_*d*_ ←0, All contents counter;

$L^{•},\bar{L}$ ←0, Un-duplicated, and Duplicated content counter, respectively;

$Sim_{th}^{sh}$ ← The similarity threshold;

Create $C_{CoD_{d}}^{L_{d}\times \kappa }$ by adding $G_{∁}^{\omega \times \kappa }$;

**for** (*j* = 1, *j* ≤ *M*, *j*++) **do**

 **for** (*l* = 1, *l* ≤ *ω*, *l*++) **do**

  Create new row in $C_{CoD_{d}}^{L_{d}\times \kappa }$;

  Add $C_{B_{j},d^{l}}$ and its Attributes $P_{B_{j},d^{l},\kappa }$ to $C_{CoD_{d}}^{L_{d}\times \kappa }$ (13);

  *L*_*d*_++;

 **end**

**end**

MBS created $C_{CoD_{d}}^{L_{d}\times \kappa }$ (13) ← the distributed cached contents list;

**Step 2: Filtering similar contents**

**for** (*y* = 1, *y* ≤ *L*_*d*_, *y*++) **do**

 $DC_{d}^{\bar{t}}$ ← Temporary list of duplicate contents;

 **for** (*y*^•^ = *y* + 1, *y*^•^ ≤ *L*_*d*_, *y*^•^++) **do**

  *dissim*(*y*, *y*^•^) ← 0;

  Clear *DC*^(*T*+1)×*K*^;

  **for** (*q* = 1, *q* ≤ *κ*, *q*++) **do**

   **if** ($P_{y}^{\left(P_{d}\right)}$ ∣∣ $P_{y^{•}}^{\left(P_{d}\right)}$ = *Null*) ∣∣ ($P_{y}^{\left(P_{d}\right)}$ ∣∣ $P_{y^{•}}^{\left(P_{d}\right)}$ *= 0)* & ($P_{d}$ *is Asymmetric)* **then**

    $\delta_{y,y^{•}}^{\left(P_{d}\right)}$ = 0

   **else**

    $\delta_{y,y^{•}}^{\left(P_{d}\right)}$ = 1

   **end**

   Add $Dis_{y,y^{•}}^{\left(P_{d}\right)}$ ⇒ *Dissim*(*y*, *y*^•^);

  **end**

  Compute Dissimilarity *dissim*(*y*, *y*^•^) according to (14);

  *sim*(*y*, *y*^•^) ← Calculate Similarity according to (15);

  Add *sim*(*y*, *y*^•^) ⇒ $SIM^{L_{d}\times \kappa }$ (16);

  **Step 3: Validity of cached contents (MBS + SBSs)**

  **if** (*sim*(*y*, *y*^•^)> = $sim_{th}^{sh}$) **then**

   $\vee^{C_{Dis_{y}}^{L_{d}\times \kappa }}$ ← Compute AcVP according to (18);

   Add $Dis_{y}^{L_{d}\times \kappa }$ to $DC_{d}^{\bar{t}}$;

   $\vee^{C_{Dis_{y^{•}}}^{L_{d}\times \kappa }}$ ← Compute AcVP according to (18);

   Add $Dis_{y^{•}}^{L_{d}\times \kappa }$ to $DC_{d}^{\bar{t}}$;

  **end**

 **end**

 **Step 4: The Final List Generation**

 Add Max $\left(DC_{d}^{\bar{t}}\right)$ ⇒ *UDCL*^*L*^•^×*κ*^ (20);

 *L*^•^++;

 Add others $\left(DC_{d}^{\bar{t}}\right)$ ⇒ $DCL^{\bar{L}\times \kappa }$ (21);

 $\bar{L}$ = $\bar{L}$ + Count($DC_{d}^{\bar{t}}$)—1;

 Clear $DC_{d}^{\bar{t}}$;

**end**

**Step 5: Duplication elimination in the Cache.**

**for** (*l* = 1, $l\leq \bar{L}$, *l*++) **do**

 **for** (*j* = 1, *j* ≤ *M*, *j*++) **do**

  **if** $H{\left(DCL^{\bar{L}\times \kappa }\right)}_{d^{l}}$ ∈ ($C_{B_{j}}^{\omega \times \kappa }$) **then**

   Remove ($C_{B_{j},d^{l}}$) (as $victim(\vee^{C_{B_{j},d^{l}}},C_{B_{j}},\bar{T})$);

  **end**

 **end**

Each SBS *B*_*j*_ broadcast UDCL to all serving MSs *U*_*j*,*i*_.

**Return** *UDCL* & *DCL*.

#### 3.1.7 Complexity of the proposed algorithm

As mentioned and discussed earlier, the total number of distributed caches represented by *M*^•^, where each cache stores *ω* contents, resulting in a total of *L* contents. Each content is characterized by *κ* attributes. The un-duplicated and duplicated contents among the distributed caches are denoted as *L*^•^ and $\bar{L}$, respectively.

Given these parameters, the complexity of the main operations in the iterative process can determine as follows:

- The processing of cached contents at the distributed caches has a time complexity of *O*(*M*^•^.*ω*).
- Filtering similar contents by performing attribute matching among the contents requires a complexity of *O*(*L*^2^).
- Eliminating duplicate contents from the target distributed cache has a time complexity of $O(\bar{L})$.

Therefore, the overall time complexity of the algorithm can be expressed as $O\left(M^{•}.\omega \right)+O\left(L^{2}\right)+O\left(\bar{L}\right)$. It is worth noting that *L* = (*M*^•^.*ω*), but $O\left(L^{2}\right)>O\left(\bar{L}\right)$ and *O*(*L*^2^)>*O*(*M*^•^.*ω*). Consequently, the overall complexity is determined as *O*(*L*^2^) = *O*((*M*^•^.*ω*)^2^).

### 3.2 Re-Distribute Un-Duplicated Cached Content (RUCC)

Based on the previous discussion, the duplicate cached contents were eliminated from the target distributed cache. For instance, some caches have more free space and others less based on the total size of the cached contents which eliminated from each cache. To gain appropriate free space in each cache with the objective of caching the new contents. In addition to avoiding the repeating of execution of the cache replacement policy (CRP), which may be evicting some of the popular contents from that cache. That results in increasing the energy consumption and delay of contents delivery. Un-duplicated distributed cached contents with medium and large sizes are required for segmenting into smaller sizes for redistributing and storing distributively.

#### 3.2.1 Proposed RUCC scheme

To do that, the *Re-Distribute Un-Duplicated Cached Content (RUCC)* scheme is proposed as shown in Algorithm 2. The *RUCC* is performed in 4 major steps, Step 1, determine the target cached contents, which their size *S*(*D*) exceeding or equal to the content verification size *V*_*S*(*D*)_ for the total cache’s size. Step 2, determine the target caches, which have a free space exceeding or equal to the cache verification size $V_{C_{B_{j}}}$ of the total cache’s size. Step 3, segment each content into smaller sizes based on the available free space in the local SBS and corresponding SBSs. Finally, the Re-distribute and store that segments distributively in step 4.

**Algorithm 2** Re-Distribute Un-Duplicated Cached Content (RUCC)

**Input:** *UDCL*^*T*×*κ*^ (20), $S(C_{B_{j}})$, $C_{B_{j}}^{f_{S}}$.

**Output:** *Seg*_*Map*_ = {$Seg_{Map}^{d^{1}}$,…,$Seg_{Map}^{d^{l}}$: 1≤ l ≤ S}.

*ω*•: Number of un-duplicated cached contents.

Seg[j,l]: List of the target contents ∈*S*(*D*).

F [j $C_{B_{j}}^{f_{S}}$]: List of the value of the free space of SBSs caches.

**Step 1: Determine Target Cached Contents.**

S = 0; counter of the target cached contents;

*V*_*S*(*D*)_ = 5% of $S_{C_{B_{j}}}$: Define value of the content verification size;

**for** (*j* = 1, *j* ≤ *M*, *j*++) **do**

 **for** *l* = 1,*l* ≤ *ω*^•^,*l*++ **do**

  **if** (*S*($C_{B_{j},d^{l}}$) ≥ *V*_*S*(*D*)_) **then**

   Add *H*($C_{B_{j},d^{l}}$) ⇒ Seg[j,l];

   S++;

  **end**

 **end**

**end**

**Step 2: Determine Target Caches.**

$V_{C_{B_{j}}}=40\%$ of $S(C_{B_{j}})$: Define value of the cache verification size;

Z ← 0: Counter of the target caches

**for** (*j* = 1, *j* ≤ *M*, *j*++) **do**

 Compute $C_{B_{j}}^{f_{S}}$ using (3);

 **if** ($C_{B_{j}}^{f_{S}}$ ≥ $V_{C_{B_{j}}}$ **then**

  Add *B*_*j*_ & $C_{B_{j}}^{f_{S}}$ ⇒ F[j $C_{B_{j}}^{f_{S}}$];

  Z++;

 **end**

**end**

**Step 3: Segment Each Target Content into Smaller Sizes.**

Determine the number of segments according to the $C_{B_{j}}^{f_{S}}$ & Z;

Q = Z: Total Number of Segments;

$S_{q}=\frac{1}{Q}$ : Segment Size;

**for** (*s* = 1, *s* ≤ *S*, *s*++) **do**

 $C_{B_{j},d^{s}}=\{{\left(C_{B_{j},d^{s}}\right)}_{1},{\left(C_{B_{j},d^{s}}\right)}_{2},\ldots ,{\left(C_{B_{j},d^{s}}\right)}_{Q}\}$ ;

 **for** (*j* = 1, *j* ≤ *Z*, *j*++) **do**

  **if** (*B*_*j*_ ∈ F[*j* $C_{B_{j}}^{f_{S}}$]) **then**

   Local SBS send the $C_{B_{j},d^{s}}$ or set of segments to *B*_*j*_;

  **end**

  **Step 4: Re-distribute & Storing the Segments Distributively.**

  Received the $C_{B_{j},d^{s}}$ or set of segments in *B*_*j*_;

  Cache the $C_{B_{j},d^{s}}$ or set of segments in *B*_*j*_;

 **end**

 Create $Seg_{Map}^{d^{l}}$;

**end**

**Return** *Seg*_*Map*_ = {$Seg_{Map}^{d^{1}}$,…,$Seg_{Map}^{d^{l}}$: 1≤ l ≤ S}.

#### 3.2.2 Complexity analysis of the RUCC schemes

As mentioned, and discussed in (3.2) and algorithm (2), the number of distributed cache is presented by *M* (only caches of SBSs). As well as, the un-duplicated which segmented is *L*^•^, where each cache cached *ω*^•^ un-duplicated contents. The total un-duplicated contents, which will be segmented is equal to *S*, where each content will be fragmented into a smaller size equal to *Q* to be cached at *Z* caches form *M*, where *Z* < = *M*. The complexity is counted as: The iterative of determining the target contents has time complexity a *O*(*M*.*ω*^•^). The iterative of determining the target caches has time complexity *O*(*M*). The iterative of determining the segmentation of the target contents and caching their segments in the target caches has time complexity *O*(*S*.*Z*). Therefore, the overall complexity of the *RUCC* is *O*(*M*.*ω*^•^) + *O*(*M*) + *O*(*S*.*Z*), where *O*(*M*.*ω*^•^) > *O*(*M*), and *O*(*M*) < *O*(*S*.*Z*). Then, the RUCC has time complexity *O*(*M*.*ω*^•^).

## 4 Experimental design and evaluation

In this work, the experiment is presented to evaluate the benefits of the UDCL for the MSs, in addition, to evaluating the benefits of redistributed the un-duplicated cached contents among the distributed cache. Then, the *Proposed Scheme* is compared with the existing schemes such as uplink with no-caching *(No-cache)*, cache assisted uplink *(Each-cache)* [15], and uplink with collaborative distribution caching *(SBS-CoDc)* [16]. The comparison is performed using different scenarios based on the number of distributed cached contents, cached content popularity, and time slots, in addition, the number of MSs. Table 2 is shown the simulation parameters.

**Table 2:** Simulation parameters.

Parameter name	Value
Coverage area	1*km*^2^
Cache capacity of MBS’s cache	In the Range 100–512 GB (128 GB)
Number of SBS	20
Coverage area of an SBS	50 meters
The Maximum distance of an SBS from MBS	100 to 500 meters
The capacity of an SBS’ cache	In Range 32–128 GB (32 GB)
Total distributed cached contents	In the Range of 250–2000
Total cached contents of a cache	In the Range of 50–500
Number of MSs	150, 300, 450, 600, 750, 900
Content size in MB	In the Range 100–999
Content size in GB	In the Range [1,…,3]
Content popularity	In the Range [5,…,100] each 5
Specific time (time slot)	In the Range [250,…,2000] each 250

### 4.1 Performance metrics

The experiment was evaluated the distributed cache and MSs using different measurements as the following:

#### 4.1.1 Performance measurements of mobile stations

The performance of the MSs were evaluated using the average uplink throughput of MSs, in addition, the following measurements:

*Average Uplink Throughput TH_avg_*. The *TH*_*avg*_ of MSs is computed according to (7) as,

$$\begin{array}{c} TH_{avg}=\frac{\sum_{i=1}^{N}{ℜ}_{U_{j,i}}^{ul}}{N} \end{array}$$ (22)

*Average energy efficiency*. The EE is the ratio of the data rate and energy consumption. Therefore, the average EE is computed by dividing the average uplink data rate of MSs referring in (22) by the average Energy consumption $Avg_{EC}^{U}$ according to (10) as,

$$\begin{array}{c} EE_{U}=\frac{TH_{avg}}{Avg_{EC}^{U}} \end{array}$$ (23)

*Average spectral efficiency*. The SE is a measure that relates the uplink capacity ℜ^*UL*^ to the total available bandwidth *B*. It quantifies the efficiency of utilizing the available spectrum for transmitting data in the uplink direction. The relationship between SE, ℜ^*UL*^, and *B* is provided in [16, 32–34] as follows:

$$\begin{array}{c} SE=\frac{{ℜ}^{UL}}{B}. \end{array}$$ (24)

According to the Shannon channel capacity, the maximum bit rate is given by (7). Then, inserting (7) into (24), the average SE can be defined as,

$$\begin{array}{c} SE_{U_{j,i}}=\frac{B\;{\text{log}}_{2}\left(1+SINR_{U_{j,i}}\right)}{B},\;then,\;SE_{U_{j,i}}={\text{log}}_{2}\left(1+SINR_{U_{j,i}}\right). \end{array}$$ (25)

Therefore, the average SE of the *N* MSs can be expressed as,

$$\begin{array}{c} SE_{U}=\frac{\sum_{i=1}^{N}SE_{U_{j,i}}}{N}. \end{array}$$ (26)

#### 4.1.2 Performance measurements of distributed cache

The performance of the SBSs caches was evaluated using the cache hit ratio (referring in (4)) and the cache hit probability (referring in (6)). Additionally, the performance of the distributed cache is evaluated using the following metrics:

*Cache diversity*. To evaluate the number of distinct cached contents, and counter the duplicate contents at the distributed cache, the cache diversity is presented. It is the ratio between the cardinality of unique contents cached in distributed cache $\left(Card{⋃}_{j=1}^{M}D_{l,j}\right)$ and the cardinality of total number of contents in the distributed cache (*CardD*_*l*,*j*_). It is taken value between $\left[\frac{1}{|M^{•}|},1\right]$. The cache diversity is denoted by *C*_*Diversity*_, and is given as by [35, 36].

$$\begin{array}{c} C_{Diversity}=\frac{Card{⋃}_{j=1}^{M^{•}}D_{l,j}}{\sum_{j=1}^{M^{•}}CardD_{l,j}}, \end{array}$$ (27)

where *M*^•^ = *M* + 1 is the total number of distributed cache in the distributed network. *l* is the serial number of the distributed cached content. *D* is the distributed cached content.

*Overall Cache Efficiency (OCE)*. The Overall Cache Efficiency (OCE) is a metric that quantifies the ratio of the cumulative cache hits to the cumulative demands. It provides an indication of the overall effectiveness of the cache in serving requested content. The OCE, denoted by *C*_*OCE*_, and is calculated based on the formula provided in reference [37].

$$\begin{array}{c} C_{OCE}=\frac{\sum_{j=1}^{M}Hit_{r}^{C_{B_{j}}}}{S_{d}}, \end{array}$$ (28)

where $\sum_{j=i}^{M}Hit_{r}^{C_{B_{j}}}$ is the cumulative of the distributed cache hit ratio. *S*_*d*_ is cumulative of the demands.

## 5 Numerical results

The evaluation of the performance of both the distributed cache of the SBSs and MSs is presented in this section.

### 5.1 Numerical results of distributed cache performance

The evaluation of the impact of the segmentation and redistribute cached contents on the distributed cache are presented.

#### 5.1.1 Distributed cache hit ratio

**Fig 2:**
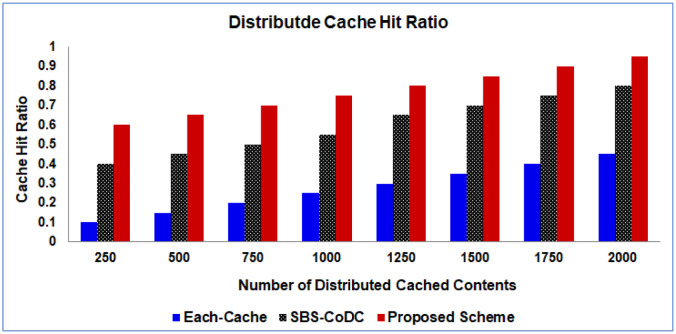
Comparison average distributed cache hit ratio.

Fig 2 shows the average of the hit ratio of distributed cache (the Each-cache, and SBS-CoDc) along with the proposed scheme.

Because of noncooperation between the Small Base Stations (SBSs), the average of the cache hit ratio of SBS-CoDc shows only a slight increase compared to Each-cache. In Each-Cache, the cache hit ratio is calculated individually for each cache and then summed. However, the proposed scheme surpasses SBS-CoDc by achieving a remarkable 29.17% improvement in the cache hit ratio. This improvement is attributed to the distribution of contents among different SBSs, coupled with the use of the Unified Caching Locator of the UDCL that acts as a map for Mobile Stations (MSs) to locate the cached contents. These enhancements have a significant impact on reducing the traffic load on the access network and backhaul links, resulting in improved EE and SE along with increasing the cache hit ratio of the distributed cache.

#### 5.1.2 Distributed cache hit probability

According to (6), the distributed cache hit probability is evaluated based on the content popularity and content caching probability. The outmatch of the proposed scheme on the SBS-CoDc by improving the cache hit probability by 74.89%.

**Fig 3:**
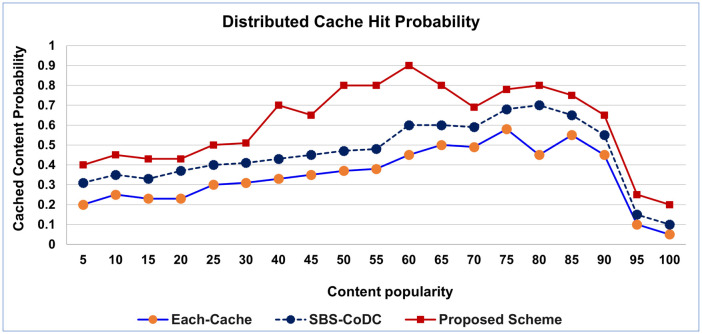
Distributed cache hit probability.

Simulation results in Fig 3 are based on increasing the value of the popularity of content. Fig 3 shows an increase in content caching probability because the segmentation and distributively caching the contents increased the availability of the contents in the all caches.

That increased the indexing of the popular content, in addition the contents with a high popularity rank increased the probability of finding the request of the mobile from any distributed cache. That increases the cache hit probability. Consequently, the curves of the proposed scheme are not stable because the value of the popularity is calculated on average based on the total number of the contents of distributed cache, which have either the same popularity or in the range as (80–85).

#### 5.1.3 Overall Distributed Cache Efficiency (OCE)

**Fig 4:**
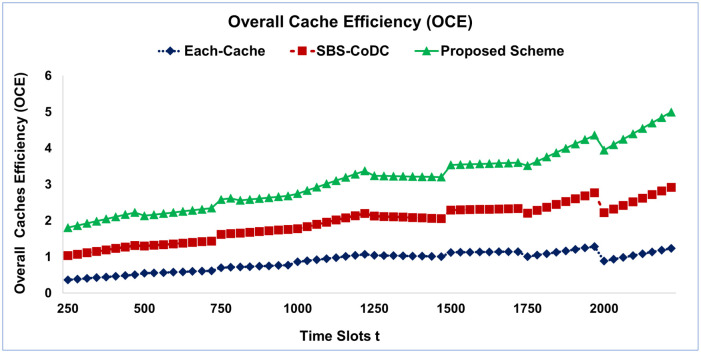
Overall distributed cache efficiency among existing schemes.

According to (28), Fig 4 shows the OCE of the distributed cache setting with all distributed cache under the impact of the proposed scheme, Each-Cache, and SBS-CoDc.

The seen that the OCE of the proposed scheme rises the fastest among the existing schemes. Until *t* = 800, the distributed caching entity received a total of 517 requests in average, and the OCE of the proposed scheme, Each-cache, and SBS-CoDc are 65, 102, and 98, respectively, and so on. A higher OCE implicates that MSs can acquire more favorite content from the distributed cache. As a result, the successful distributed cache hits are 450, 800, and 950, by Each-Cache, SBS-CoDc, and proposed scheme, respectively. More specifically, the proposed scheme participates in more than 19% and 16% OCE, compared with the Each-Cache, and SBS-CoDc, respectively. The reason behind that, the setting of the distributed cache has an optimal cache, and a limited cache cannot store all the cached contents with large or medium sizes, in addition, the most popular contents are stored distributively. For that, some time slots were taken by the proposed scheme to obtain enough decisions of each mobile so that to infer mobile’s dependency to the target distributed cache based on the availability of contents. Furthermore, the OCE of the proposed scheme increased with an increase of time slots *t*.

#### 5.1.4 Distributed cache diversity

**Fig 5:**
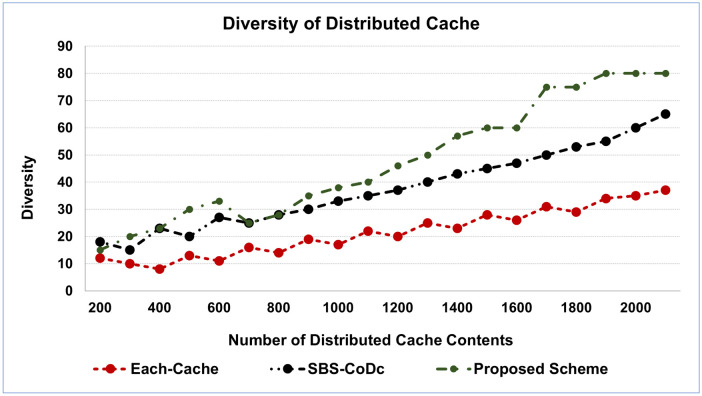
Diversity of distributed cache.

**Fig 6:**
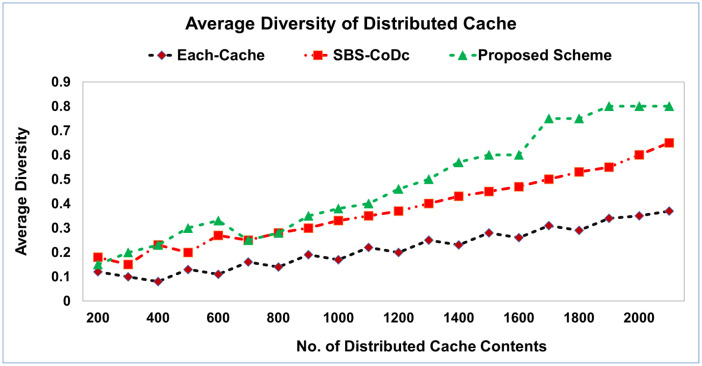
Average of diversity of distributed cache.

To evaluate eliminating the duplicate contents between the distributed cache, the cache diversity is presented to show the similar contents among the distributed cache, which is eliminated. Fig 5 shows the distributed cache diversity, while Fig 6 shows the average diversity of distributed cache.

The proposed scheme shows a high diversity of caches. This is because the matching and determining the similarity is performed among the contents of all the distributed cache. That generated the replicas of the same contents, which belong to the different caches. Furthermore, the diversity increased when the number of cached contents increases. This is because there are different MSs uploading or requesting for different contents overall the network. The diversity of the proposed scheme can significantly reduce expected run times by utilizing the searching contents within the distributed cache, in addition, optimizing the contents distributed among the distributed cache. That achieved more benefits for the MSs such as more free space in the distributed cache and reduce the cost of the transmissions. That resulted in the distributed cache being more efficient.

### 5.2 Numerical results of mobiles performance

The performance of the MSs under impact of the UDCL is evaluated in this subsection.

#### 5.2.1 Improvement of Uplink Throughput of MSs

**Fig 7:**
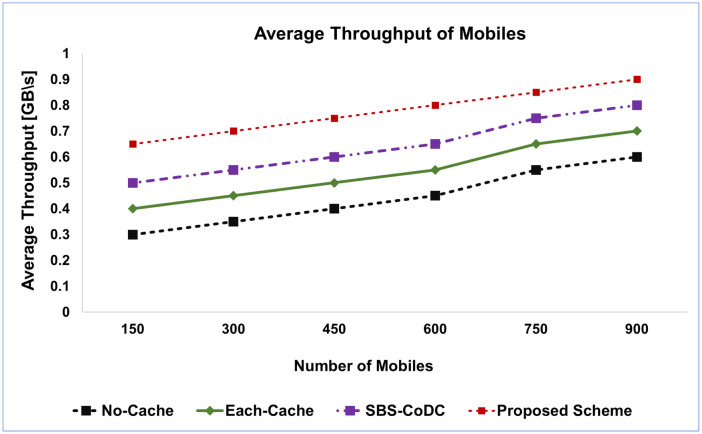
Comparison average of throughput of MSs.

The average Uplink Throughput (**TH**) of the proposed scheme is compared with the existing schemes as shown in Fig 7. The proposed scheme has improved the **TH** almost by 76.67%, 29.27%, and 17.78% as compared to No-Cache, Each-Cache, and SBS-CoDc, respectively.

The proposed scheme demonstrates significantly improved throughput (**TH**) for MS compared to the No-Cache scheme, which is expected due to the utilization of caching. Similarly, when compared to Each-Cache, the proposed scheme outperforms it due to its distributed nature. The results clearly indicate that the proposed scheme surpasses SBS-CoDc by leveraging segmentation and redistribution of contents, as opposed to storing the entire content in a cache. Additionally, unlike existing schemes, the proposed approach performs content matching at the MS level rather than the SBS level. This eliminates the need for content upload and leads to a substantial enhancement in throughput.

#### 5.2.2 Improved MS’s energy efficiency

**Fig 8:**
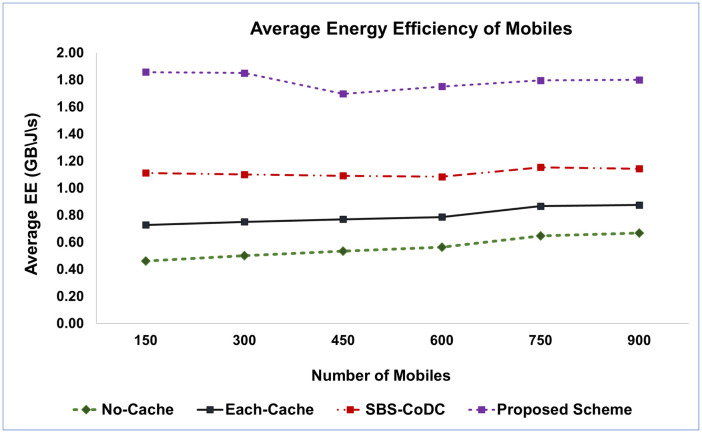
Comparison average EE of MSs among existing schemes.

Energy efficiency (EE) serves as a valuable metric for assessing performance enhancements. As observed in previous studies, lower energy consumption typically correlates with improved EE. In this research, the proposed scheme is evaluated and compared to existing schemes based on EE. Fig 8 illustrates the EE of an MS as the number of MSs increases.

Referring to Eq (23), the findings depicted in Fig 8 affirm that the average Energy Efficiency (EE) of the proposed scheme surpasses that of existing schemes. In comparison to SBS-CoDc, the proposed scheme exhibits a remarkable improvement in EE, reaching an impressive 78%. This notable enhancement can be attributed to the utilization of the Unified Caching Locator of the UDCL for content matching at the MS level, resulting in a reduction in content uploads when a cache hit occurs. Furthermore, the proposed scheme outperforms other existing schemes by significantly enhancing the average EE by 46% in contrast to SBS-CoDc. This substantial improvement is primarily attributed to the improved hit ratio achieved through the segmentation and redistribution of contents, which eliminates the need to store the entire content in a cache.

#### 5.2.3 Improvement in Spectral Efficiency (SE) of MSs

**Fig 9:**
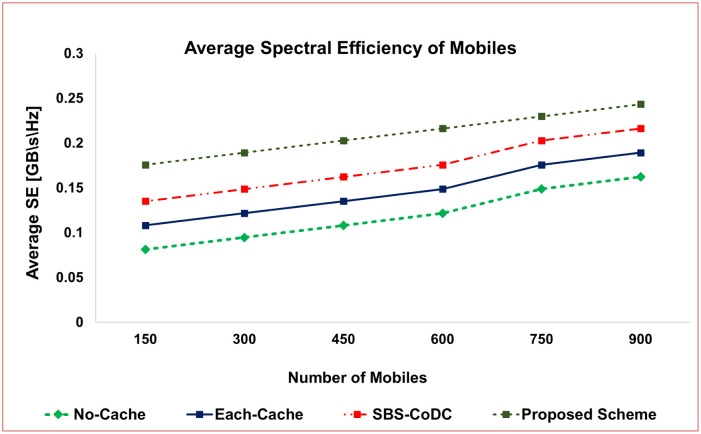
Comparison average spectral efficiency of MSs.

Referring to Eq (26), Fig 9 illustrates the average SE of MSs compared to the No-cache, Each-cache, SBS-CoDc, and the proposed scheme for varying numbers of MSs.

The proposed scheme exhibits a notable enhancement in Spectral Efficiency (SE), achieving an improvement of approximately 18% compared to SBS-CoDc. Moreover, when compared to Each-Cache, the improvement reaches an impressive 29.27%. The improved SE can be attributed to a significant reduction in the number of uplink contents. The decision-making process at the MS level regarding content upload plays a crucial role. When a cache hit occurs, the contents are not uploaded, effectively conserving bandwidth for other requests from the remaining MSs. Consequently, a substantial amount of spectrum is saved, enabling the accommodation of more requests and ultimately leading to an improved SE.

## 6 Conclusion

This paper presented efficient uplink cache schemes in a distributed scenario. The proposed schemes improved energy and spectral efficiency by leveraging content matching among distributed cache, resulting in increased free space and cache hit ratio. Local content matching has reduced the duplicate contents, while providing the MS with a list of cached contents to improve the cache hit ratio. Consequently, the schemes enhanced throughput, EE, and SE of the access network. In rare cases where free space in a distributed cache is insufficient for new content, the distributed cache performed content replacement. Segmentation and redistribution of cached contents with large or medium sizes further improved cache hit ratio, probability, and Overall Cache Efficiency (OCE). Analysis demonstrated that the proposed schemes outperformed existing schemes by increasing cache hit ratio, probability, OCE, and diversity by 29.17%, 74.89%, 24.17%, and 80%, respectively. Moreover, the scheme enhanced **TH**, SE, and EE of the access network by 17.78%, 18%, and 78%, respectively, and improved EE and SE of the sidehaul and backhaul links of the SBSs.

## Supporting information

S1 File (ZIP)

S2 File (ZIP)

S3 File (ZIP)

S4 File (ZIP)

S5 File (RAR)
